# N6‐methyladenosine reader YTHDF3 regulates melanoma metastasis via its ‘executor'LOXL3

**DOI:** 10.1002/ctm2.1075

**Published:** 2022-11-02

**Authors:** Hao‐ze Shi, Jing‐shu Xiong, Lu Gan, Ying Zhang, Cong‐cong Zhang, Ying‐qi Kong, Qiu‐ju Miao, Cui‐cui Tian, Rong Li, Jin‐quan Liu, Er‐jia Zhang, Wen‐bo Bu, Yan Wang, Xian‐feng Cheng, Jian‐fang Sun, Hao Chen

**Affiliations:** ^1^ Department of Pathology, Institute of Dermatology Chinese Academy of Medical Sciences and Peking Union Medical College Nanjing China; ^2^ Laboratory of Mycobacteriology, Institute of Dermatology Chinese Academy of Medical Sciences and Peking Union Medical College Nanjing China; ^3^ Department of Sexually Transmitted Disease, Institute of Dermatology Chinese Academy of Medical Sciences and Peking Union Medical College Nanjing China; ^4^ Department of Physiotherapy, Institute of Dermatology Chinese Academy of Medical Sciences and Peking Union Medical College Nanjing China; ^5^ National Center for STD Control China CDC Nanjing China; ^6^ Department of Dermatology China Aerospace Science & Industry Corporation 731 Hospital Beijing China; ^7^ Department of Surgery, Institute of Dermatology Chinese Academy of Medical Sciences and Peking Union Medical College Nanjing China; ^8^ Department of Clinical Laboratory, Institute of Dermatology Chinese Academy of Medical Sciences and Peking Union Medical College Nanjing China

**Keywords:** Lysyl oxidase‐like 3 (LOXL3), melanoma, metastasis, N6‐methyladenosine, YTH N6‐methyladenosine RNA binding protein 3 (YTHDF3)

## Abstract

**Background:**

A number of studies have demonstrated that N6‐methyladenosine (m6A) plays a vital role in the pathological process of various tumours. Recently, it was found that m6A writers or erasers affect the tumourigenesis of melanoma. However, the relationship between m6A readers such as YTH domain family (YTHDF) proteins and melanoma was still elusive.

**Methods:**

RT‐qPCR, Western blot and immunohistochemistry were conducted to measure the expression level of YTH N6–methyladenosine RNA binding protein 3 (YTHDF3) and lysyl oxidase–like 3 (LOXL3) in melanoma tissues and cells. The effects of YTHDF3 and LOXL3 on melanoma were verified in vitro and in vivo. Multi‐omics analysis including RNA‐seq, MeRIP‐seq, RIP‐seq and mass spectrometry analyses was performed to identify the target. The interaction between YTHDF3 and LOXL3 was verified by RT‐PCR, Western blot, MeRIP‐qPCR, RIP‐qPCR and CRISPR‐Cas13b‐based epitranscriptome engineering.

**Results:**

In this study, we found that m6A reader YTHDF3 could affect the metastasis of melanoma both in vitro and in vivo. The downstream targets of YTHDF3, such as LOXL3, phosphodiesterase 3A (PDE3A) and chromodomain helicase DNA‐binding protein 7 (CHD7) were identified by means of RNA‐seq, MeRIP‐seq, RIP‐seq and mass spectrometry analyses. Besides, RT‐qPCR, Western blot, RIP‐qPCR and MeRIP‐qPCR were performed for subsequent validation. Among various targets of YTHDF3, LOXL3 was found to be the optimal target of YTHDF3. With the application of CRISPR–Cas13b‐based epitranscriptome engineering, we further confirmed that the transcript of LOXL3 was captured and regulated by YTHDF3 via m6A binding sites. YTHDF3 augmented the protein expression of LOXL3 without affecting its mRNA level via the enrichment of eukaryotic translation initiation factor 3 subunit A (eIF3A) on the transcript of LOXL3. LOXL3 downregulation inhibited the metastatic ability of melanoma cells, and overexpression of LOXL3 ameliorated the inhibition of melanoma metastasis caused by YTHDF3 downregulation.

**Conclusions:**

The YTHDF3‐LOXL3 axis could serve as a promising target to be interfered with to inhibit the metastasis of melanoma.

## Graphical abstract

YTHDF3 regulates migration, invasion and metastasis of melanoma cells both in vitro and in vivo. This function is related to LOXL3, a potential ‘executor’ of YTHDF3. Mechanistically, YTHDF3 recognizes the m6A sites on LOXL3 and promotes its protein expression without affecting its mRNA level via enrichment of eIF3A on the transcript of LOXL3.

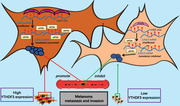

## 1 INTRODUCTION

N6‐methyladenosine (m6A) is a common methylation modification in eukaryotic mRNA, which is involved in the occurrence and development of various tumours and affects the splicing, decay, export and translation of RNA.^1^ Currently, studies focusing on the interaction between m6A and tumourigenesis have been widely reported.^2^, ^3^, ^4^, ^5^, ^6^, ^7^ However, reports associated with m6A, especially in melanoma were mainly focused on m6A writers and erasers,^8^, ^9^, ^10^, ^11^, ^12^, ^13^, ^14^, ^15^, ^16^, ^17^ only one report demonstrated that YTHDF3‐CTNNB1 axis could affect the biological process of ocular melanoma.^18^ More specific role of m6A readers like YTHDF3 in tumourigenesis of melanoma, especially cutaneous melanoma, was elusive.

Melanoma is the most malignant skin tumour. The low average survival rate of patients is caused by their high malignant features, metastatic ability and insensitivity to radiotherapy or chemotherapy. Moreover, the treatment of metastatic melanoma is a major clinical challenge. At present, new drugs mainly include molecular targeting drugs and immune checkpoint inhibitors, such as BRAF*^V600E^* inhibitors^19^ and anti‐PD‐1 antibodies.^20^ Compared with traditional drugs, these drugs have better efficacy, but the problems of drug resistance and low efficacy make it necessary to search for new therapeutic targets and drugs.

In this study, we found that YTHDF3 was highly expressed in melanoma tissues and cell lines compared with benign nevi samples or epidermal melanocytes. Also, we confirmed that YTHDF3 has an impact on the metastasis of melanoma both in vitro and in vivo. The underlying mechanism and downstream target of such an effect was then identified by the multi‐omics analysis. Among various targets identified by multi‐omics analysis, LOXL3 stood out as an optimal target of YTHDF3 in melanoma. With CRISPR–Cas13b‐based epitranscriptome engineering tool, we confirmed that the LOXL3 transcript was captured by YTHDF3 via its m6A sites. YTHDF3 could augment the protein expression of LOXL3 without affecting its mRNA level in an m6A‐dependent manner and such a phenomenon could be boiled down to eukaryotic translation initiation factor 3 subunit A (eIF3A), while the RNA stability was not affected by YTHDF3. Our study revealed the oncogenic role of YTHDF3 and its target LOXL3 in melanoma metastasis and its specific mode of action.

## 2 MATERIALS AND METHODS

### 2.1 Tissue samples collection

Samples including benign nevi and malignant melanoma were formalin‐fixed and paraffin‐embedded. All samples were collected from our hospital. Informed consent was collected from participant patients whose samples will be used for this study.

### 2.2 Cell lines and cell culture

Primary epidermal melanocytes were isolated from human samples according to the protocols of our research group. Malignant melanoma cell lines such as A375, A875, SK‐MEL‐28, M14, MV3 and SK‐MEL‐5 were preserved in our research group. A2058 cell line was kindly provided by Professor Chunying Li from Xi Jing hospital. DMEM medium was combined with 10% foetal bovine serum (FBS) and 1% penicillin and streptomycin for the culture of all melanoma cells. Above mentioned cells were incubated at the temperature of 37°C and in an atmosphere of 5% CO_2_.

### 2.3 RNA isolation and RT‐qPCR

Total RNA extraction was treated with RNAiso plus (Takara, Dalian, China) from different cells according to manufacturers’ instructions. Then, the extracted RNA was reversely transcribed to cDNA by PrimeScript^™^ RT Master Mix (Takara, RR036A, Dalian, China). Samples were then analysed with Roche LC480. Expression data were normalized to β‐actin. The 2^−ΔΔCt^ method was employed for the normalization of relative gene expression. The sequence of primers of related genes was as follows: β‐actin F:5′ GTGGCCGAGGACTTTGATTG 3′; β‐actin R :5′ CCTGTAACAACGCATCTCATATT 3′; YTHDF3 F: 5′ GGTGTATTTAGTCAACCTGGGG 3′; YTHDF3 R: 5′ AAGAGAACTAGGTGGATAGCCAT 3′; LOXL3‐F: 5′ GATACAGCGAGCTGGTGAATG 3′; LOXL3‐R: 5′ CATCCTCATCGTGCGTACAGT 3′; CHD7‐F: 5′ TGATGAGTCTTTTTGGCGAGG 3′; CHD7‐R: 5′ CTGGATTTTCCGGGTAACCAC 3′; PDE3A‐F: 5′ CCACGGCCTCATTACCGAC 3′; PDE3A‐R: 5′ TTGCTCACGGCTCTCAAGG 3′; METTL3‐F: 5′ TTGTCTCCAACCTTCCGTAGT 3′; METTL3‐R: 5′ CCAGATCAGAGAGGTGGTGTAG 3′

### 2.4 Western blotting

Combined with a protease inhibitor cocktail and phosphatase inhibitor cocktail, RIPA lysis buffer was employed to make cells lysed. Protein concentrations were measured with a BCA kit. Separated by SDS‐PAGE, the protein was transferred onto the PVDF membranes. 5% BSA was used for blocking up to 2 h, the membranes then were incubated with primary antibodies at 4°C overnight. After the incubation and the subsequent washing for 30 min, membranes were incubated with HRP Goat Anti‐Rabbit IgG or HRP Goat Anti‐Mouse IgG antibodies at room temperature. Primary antibodies were as follows: anti‐YTHDF3 (ab220161, 1:1000 dilution, Abcam), anti‐LOXL3 (sc‐377216, 1:100 dilution, Santa‐Cruz), anti‐β‐actin (#4970, 1:1000 dilution, Cell Signaling Technology), anti‐eIF3A (ab128996, 1:1000 dilution, Abcam), anti‐METTL3 (15073‐1‐AP, 1:1000 dilution, Proteintech), anti‐METTL14 (26158‐1‐AP, 1:1000 dilution, Proteintech), anti‐FTO (27226‐1‐AP, 1:1000 dilution, Proteintech), anti‐ALKBH5 (16837‐1‐AP, 1:1000 dilution, Proteintech), anti‐CHD7 (A17180, 1:1000 dilution, ABclonal), anti‐PDE3A (A17919, 1:1000 dilution, ABclonal).

### 2.5 Haematoxylin and eosin staining and immunohistochemistry (IHC) staining

A haematoxylin–eosin (H&E) staining was carried out on paraffin‐embedded sections slides, and IHC staining was performed with an autostainer (link45 System, Dako, Denmark) with anti‐YTHDF3 (ab220161, 1:200 dilution, Abcam). Slides were analysed by Ying Zhang, Jian‐fang Sun and Hao Chen from our hospital.

### 2.6 Plasmid construction and lentiviral transfection

In order to construct the lentivirus‐carried plasmid (shRNA) for targeting YTHDF3, LOXL3 and METTL3, related complementary sense and antisense oligonucleotides were synthesized and cloned into GV493 vectors (hU6‐MCS‐CBh‐gcGFP‐IRES‐puromycin). The plasmid of overexpression of LOXL3 was also produced and cloned into CV572 vectors (Ubi‐MCS‐SV40‐Cherry‐IRES‐neomycin). dCas13b‐FTO fusion protein was cloned to CV025 vectors (EF1A‐dCas13b‐FTO‐T2A‐puromycin). Guide RNA (gRNA, sgNC and sgLOXL3) were cloned into CV268 vectors (MCS‐SV40‐neomycin). The sequence of sgNC was GTAATGCCTGGCTTGTCGACGCATAGTCTG. The sgLOXL3 had three designed sequences to target different m6A site, which were CCGGTGAGGGAGAGGCTGCAGTGTGATATGGGGATGGGCA; AATGCCTGGGTTTGGCTAATAGGGTGATCTGTGTACTTTC; TGCCCTCATGTGGTGTACATCTTTTATGTACATGTTGCAC. All these lentivirus vectors or plasmids were constructed by GeneChem (Shanghai, China). A375, SK‐MEL‐28 and A2058 cells were transfected with supernatants containing lentivirus carrying the shYTHDF3, shLOXL3, shMETTL3, overexpression of LOXL3, dCas13b‐FTO, sgNC and sgLOXL3. The cells were selected with puromycin (2 μg/mL) or G418 (1000 μg/ml) after transfection for 72 h.

### 2.7 Transwell assays

Transwell assays include migration and invasion assays, which were conducted in a 24‐well transwell chamber system (Corning, USA). For the migration assay, 5 × 10^4^ cells with 0.2 ml FBS‐free DMEM were seeded into the upper chamber of the well. The wells were inserted into the lower chambers with 0.6 ml DMEM and 20% FBS. In terms of invasion, the upper chamber was first coated with Matrigel, and other steps were similar to migration assay. The membrane of the well was fixed with methanol and subsequently stained with crystal violet after 16 to 24 h of incubation. After staining, wells were washed with water and the cells on the membrane were taken with photos under a microscope.

### 2.8 Wound healing experiment

A total of 5 × 10^5^ cells were seeded in each well of a 6‐well plate and then incubated for 24 h. With the assistance of the 200 μl pipette tip, a linear wound on the surface of the cells was scratched. Then cells were incubated with FBS‐free DMEM. At 0, 24, 48, 72, the photos of the wound were taken.

### 2.9 In vivo metastasis assays

Animal experiments were followed with strict guidelines which were approved by the Institute of Dermatology, Chinese Academy of Medical Sciences and Peking Union Medical College. A375 melanoma cell was adopted to construct a nude mouse tumour metastasis model. 2 × 10^6^ cells of shNC and shYTHDF3 were suspended in 100 μl PBS and injected intravenously from the tail vein into each 5‐week‐old BALB/c nude mouse. Injected with D‐luciferin, potassium salt (15 mg/ml) after 15‐20 min, we used the IVIS Spectrum animal imaging system (Perkin Elmer) to evaluate the tumour metastasis condition after 7 weeks of injection with autoexposure time. The intensity unit was (p/sec/cm^2^/sr). Then mice were sacrificed. We collected the lungs, livers, and brains and fixed them for H&E and IHC staining.

### 2.10 RNA high‐throughput sequencing (RNA‐seq)

RNAs were extracted from the shNC and shYTHDF3 of A375 cells. RNA sequencing libraries were subsequently generated using poly(A) RNA purified with a PolyT tract mRNA isolation system. Generated libraries were then sequenced on the Illumina high‐throughput sequencing platform (NovaSeq 6000) according to the manufacturer's recommendations.

### 2.11 RNA binding protein immunoprecipitation and high‐throughput sequencing (RIP‐seq) and RIP‐qPCR

According to the manufacturer's instruction, Magna RIP^™^ RNA‐Binding Protein Immunoprecipitation Kit (Catalogue No.17‐700, Millipore) was used to perform the RIP assay. Briefly, 5 × 10^7^ targeted cells that were targeted and lysed were collected and lysed using RIP lysis buffer. Using A/G magnetic beads, the lysis was immunoprecipitated with targeted antibodies to the relevant RBP. After incubation overnight, immobilization of magnetic beads bound complexes was achieved using a magnet, and the beads were washed more than six times. The target protein was verified by Western blot and immunoprecipitated RNA was quantified by RT‐qPCR analysis and subsequent RIP‐seq.

### 2.12 m6A RNA immunoprecipitation and high‐throughput sequencing (MeRIP‐seq) and MeRIP‐qPCR

According to the manufacturer's instructions, Magna MeRIP^™^ m6A Kit (Catalogue No.17‐10499, Millipore) was used to perform the MeRIP assay. After total RNA was extracted, RNA was fragmented, and the size distribution of fragments was verified. RNA fragmentation was accomplished by immunoprecipitating with m6A antibody and IgG antibody using A/G magnetic beads. Then magnetic beads were eluted and targeted RNA was purified. Targeted RNA with abundant m6A sites was analysed by RT‐qPCR and MeRIP‐seq.

### 2.13 Mass spectrometry analysis

Following the manufacturer's protocol, mass spectrometry analysis was performed. For peptide and protein identification, the global false discovery rate (FDR) threshold was set at 0.01. Protein abundance was calculated based on normalized spectral protein intensity (LFQ intensity).

### 2.14 Gene ontology and KEGG pathway analysis

To explore the biological processes (bp), the cellular components (cc) and the molecular functions (mf) of the differentially expressed mRNAs and proteins, GO analysis (http://www.geneontology.org) was performed in RNA‐seq, RIP‐seq, MeRIP‐seq and differentially expressed protein sequences in mass spectrometry. GO package in the R environment was used to analyse these data. We used Fisher's Exact Test to determine differentially expressed RNAs and proteins associated with GO terms in order to enrich GO terms. We used the KEGG database to conduct the pathway analysis. We also used Fisher's Exact Test to determine differentially expressed RNAs and proteins correlated to GO terms in order to enrich KEGG terms.

### 2.15 Global RNA m6A quantification

A total RNA extraction was prepared as described below. The EpiQuik m6A RNA Methylation Quantification Kit (P‐9005; Epigentek Group Inc., USA) was used to quantify global m6A levels. 200 ng of RNA was used to be analysed. In each well, 450 nm light absorbance was used to measure the levels of m6A.

### 2.16 RNA stability assay

An actinomycin D concentration of 10 mg/ml was applied to A375, SK‐MEL‐28 and A2058 (shNC and shYTHDF3) cells. At specific time points (0, 1, 2, 4 and 8 h), the RNA of these cells was collected. The mRNA level of LOXL3 and YTHDF3 was measured by RT‐qPCR.

### 2.17 Protein stability and degradation assay

A375, SK‐MEL‐28 and A2058 (shNC and shYTHDF3) cells were treated with 250 μM CHX and 20 μM MG‐132. At specific time points (0, 2, 4, 8 and 12 h), the protein of these cells was collected. The protein level of LOXL3 was measured.

### 2.18 Statistical analysis

All experiments were conducted at least three times. Student's *t*‐test was adopted to analyse the difference between the two groups, and ANOVA was employed to analyse multiple groups. Asterisks like *, **, *** stand for *p* < .05, *p* < .01 and *p* < .001, respectively. ns stands for not significant (*p* > .05).

## 3 RESULTS

### 3.1 YTHDF3 is highly expressed in melanoma tissues and cell lines

**FIGURE 1:**
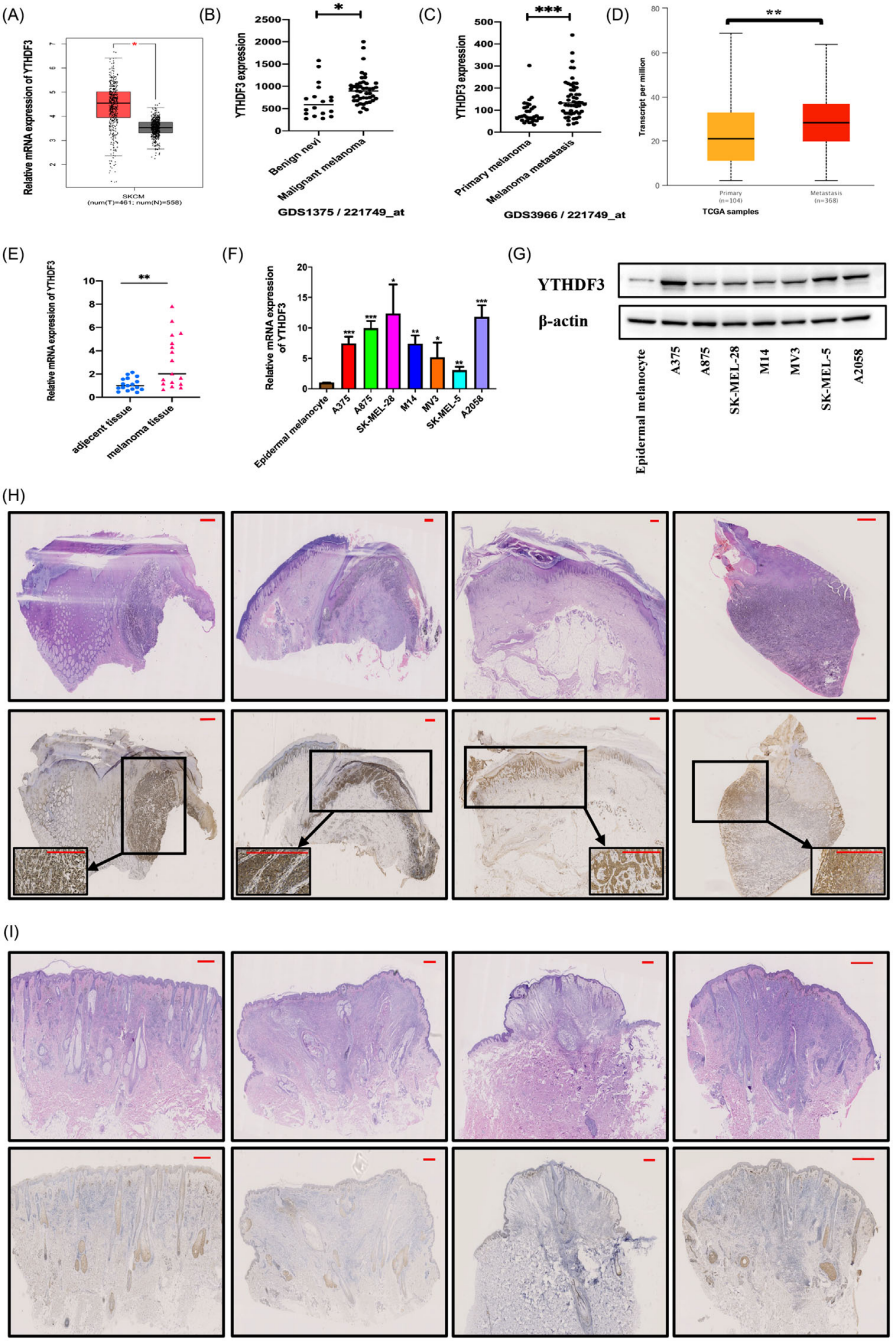
Expression of YTHDF3 is increased in melanoma. (A) mRNA expression of YTHDF3 in melanoma tissues (left, red bar) and normal tissues (right, grey bar) in the GEPIA dataset. (B) mRNA expression of YTHDF3 in benign nevi and malignant melanoma in the GEO dataset (GDS1375). (C) mRNA expression of YTHDF3 is higher in metastatic melanoma than that in primary melanoma in the GEO dataset (GDS3966). (D) mRNA expression of YTHDF3 is higher in metastatic melanoma than that in primary melanoma in the UALCAN dataset. (E) mRNA expression of YTHDF3 is higher in melanoma tissue than that in adjacent tissue (*n* = 17). (F) YTHDF3 mRNA level in epidermal melanocyte and melanoma cell lines (A375, A875, SK‐MEL‐28, M14, MV3, SK‐MEL‐5 and A2058) verified by RT‐qPCR. (G) YTHDF3 protein level in epidermal melanocyte and melanoma cell lines (A375, A875, SK‐MEL‐28, M14, MV3, SK‐MEL‐5 and A2058) verified by Western blot. (H) The YTHDF3 protein level in melanoma tissues assessed by immunohistochemistry. Scale bar = 500 μm. (I) The YTHDF3 protein level in nevi tissues assessed by immunohistochemistry. Scale bar = 500 μm. Data are shown as means ± S.D. *, ** and *** means *p* < .05, *p* < .01 and *p* < .001, respectively.

According to the online bioinformatic tools including GEPIA (http://gepia.cancer‐pku.cn),^21^ GEO dataset and UALCAN (http://ualcan.path.uab.edu),^22^ results showed that YTHDF3 was highly expressed in melanoma tissues (Figure 1A,B). The GEPIA and UALCAN database were based on the TCGA database. Besides, it was found that compared with primary melanoma, the YTHDF3 expression was higher in metastatic melanoma (Figure 1C,D). In these datasets, we also found the expression pattern of other m6A readers such as YTHDF1, YTHDF2, YTHDC1 and YTHDC2 (Figure S1A–D). Although these readers could also have a significant expression in some of these datasets, only YTHDF3 had a consistent significant expression in all datasets, indicating that YTHDF3 may play a vital role in determining the metastatic behaviour of melanoma.

We performed RT‐qPCR to compare the RNA level of YTHDF3 in melanoma tissues and adjacent tissues, epidermal melanocytes and various melanoma cell lines. We found that YTHDF3 was highly expressed in melanoma tissues (Figure 1E) and melanoma cells (Figure 1F), and, the protein level of YTHDF3 was high in these cell lines detected by Western blot (Figure 1G). Immunohistochemical results revealed that YTHDF3 was highly expressed in melanoma tissue samples (Figure 1H) compared with benign nevi tissues (Figure 1I). These findings suggested that YTHDF3 was highly expressed in melanoma tissues and cell lines and may have an impact on malignant behaviour like metastasis in melanoma.

### 3.2 YTHDF3 regulates migration and invasion of melanoma cells in vitro

**FIGURE 2:**
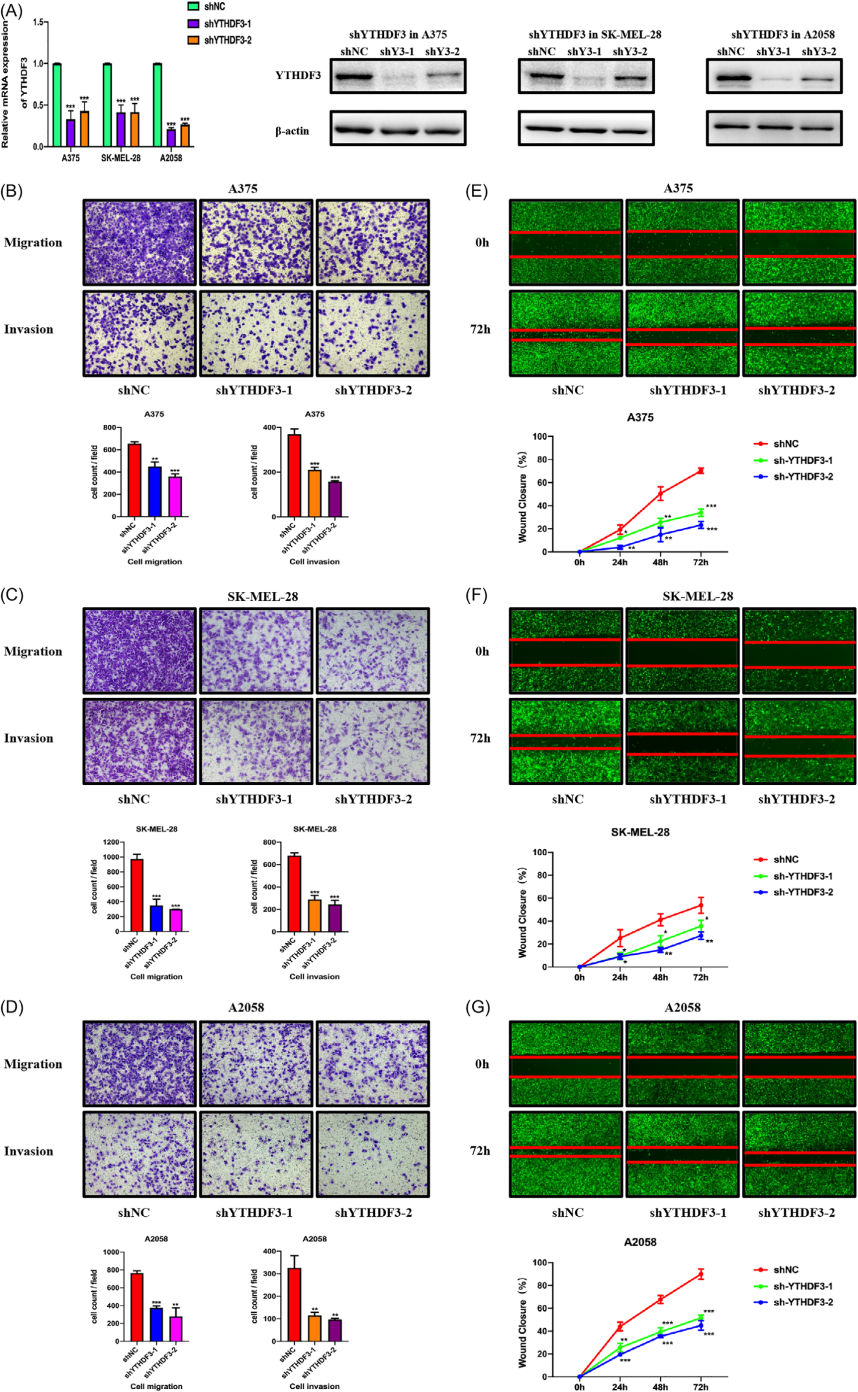
YTHDF3 downregulation inhibits melanoma cell migration, invasion and metastasis in vitro. (A) A375, SK‐MEL‐28 and A2058 cells were transfected with two shRNAs targeting YTHDF3 and a negative control shRNA. Expression of YTHDF3 mRNA and protein were detected by RT‐qPCR and Western blot. (B‐D) Transwell assay showed that YTHDF3 downregulation inhibited migration and invasion ability of A375, SK‐MEL‐28 and A2058 melanoma cells. (E–G) The wound healing experiment showed that YTHDF3 downregulation inhibited the migration ability of A375, SK‐MEL‐28 and A2058 melanoma cells in 24, 48, 72. Data are shown as means ± S.D. *, ** and *** means *p* < .05, *p* < .01 and *p* < .001, respectively.

To determine whether YTHDF3 could affect malignant behaviour such as migration and invasion of melanoma, we conducted a related experiment in vitro. With the help of shRNAs (shYTHDF3‐1 and shYTHDF3‐2), we downregulated YTHDF3 in melanoma cells including A375, SK‐MEL‐28 and A2058 cells. The efficiency of downregulation was determined by RT‐qPCR and Western blot (Figure 2A). Downregulation of YTHDF3 induced the inhibition of migration and invasion of melanoma cells determined by transwell assay (Figure 2B–D) and wound healing experiment (Figure 2E–G) in A375, SK‐MEL‐28 and A2058 cells. These results demonstrated that YTHDF3 may serve as an important molecule in regulating the migration and invasion of melanoma cells, and this phenomenon was in accordance with the enhanced expression of YTHDF3 in metastatic melanoma tissues.

### 3.3 YTHDF3 regulates metastasis of melanoma in vivo

**FIGURE 3:**
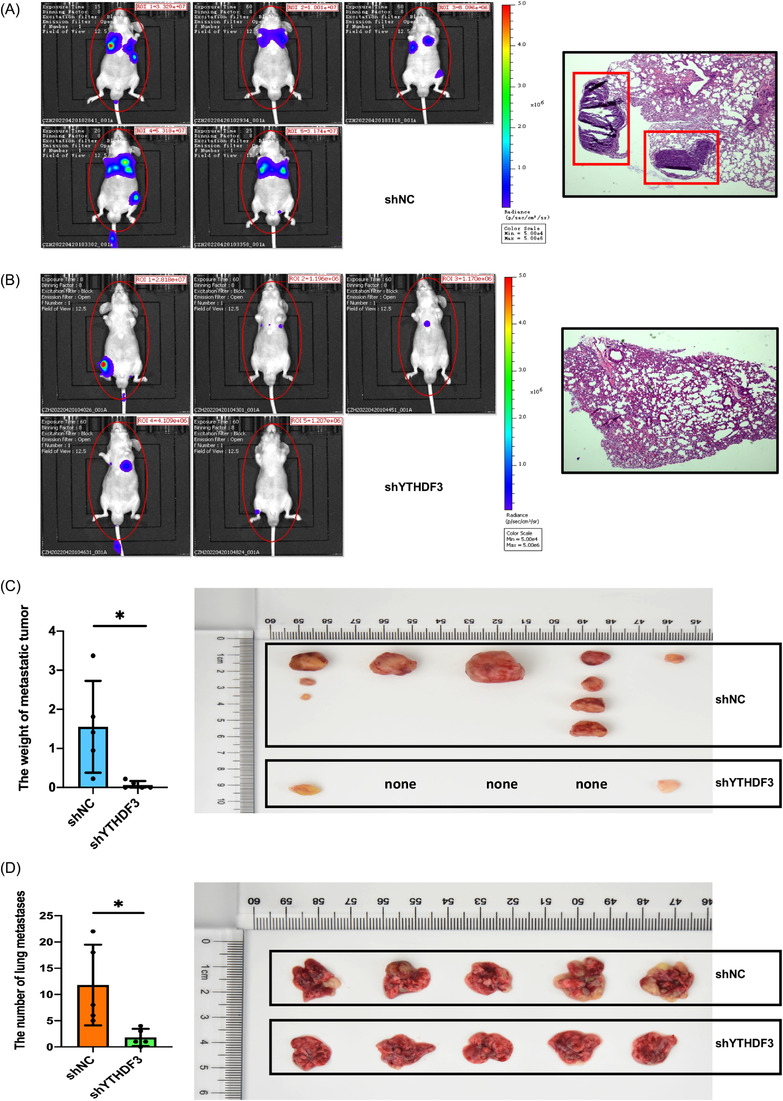
YTHDF3 downregulation inhibits melanoma metastasis in vivo. (A–D) YTHDF3 downregulation decreases subcutaneous metastatic sites and inhibited lung metastasis of melanoma cells in mice. Data are shown as means ± S.D. *, ** and *** means *p* < .05, *p* < .01 and *p* < .001, respectively.

To evaluate the function of YTHDF3 in vivo, YTHDF3‐downregulation (shYTHDF3‐1) A375 cells with luciferase expression and corresponding control shNC cells were established. Tail vein injection tumour metastatic model was performed with nude mice. The in vivo imaging system was applied to evaluate the tumour metastasis in the whole body and key organs. After 7 weeks, we observed that the mice injected with YTHDF3‐ downregulation cells got low rate of metastasis. Compared with shNC group, shYTHDF3 group had less metastatic subcutaneous tumour and lung metastases (Figure 3A–D). These results indicated that knocking down YTHDF3 could inhibit the metastasis of melanoma in vivo.

### 3.4 The downstream targets of YTHDF3 identified by multi‐omics analysis

**FIGURE 4:**
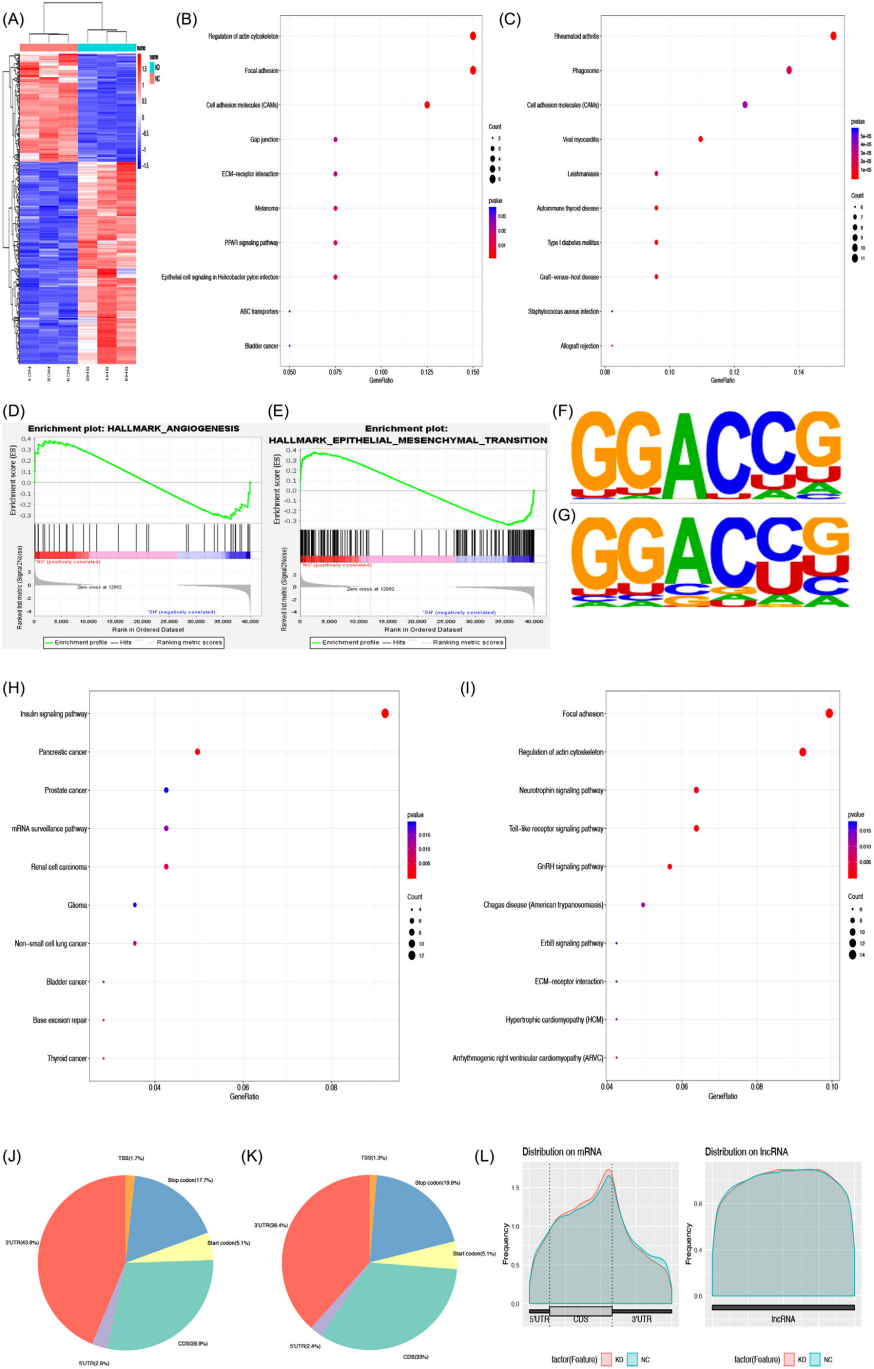
Transcript profile regulated by YTHDF3 in melanoma cells. (A) Heatmap of differentially expressed genes (DEGs) identified by RNA‐seq between shNC group and shYTHDF3 group. KEGG enrichment analysis of (B) downregulated and (C) upregulated DEGs. (D,E) Pathways of DEGs revealed by GSEA analysis. (F,G) The m6A motif detected by the MEME motif analysis. KEGG enrichment analysis of genes with differentially (H) downregulated and (I) upregulated expressed m6A level in MeRIP‐seq between shNC group and shYTHDF3 group. (J,K) Pie diagram of m6A peak distribution on RNA structure in MeRIP‐seq between shNC group and the shYTHDF3 group. (L) Metagene plot of m6A peak distribution on structures of mRNA and lncRNA in MeRIP‐seq between shNC group and shYTHDF3 group.

**FIGURE 5:**
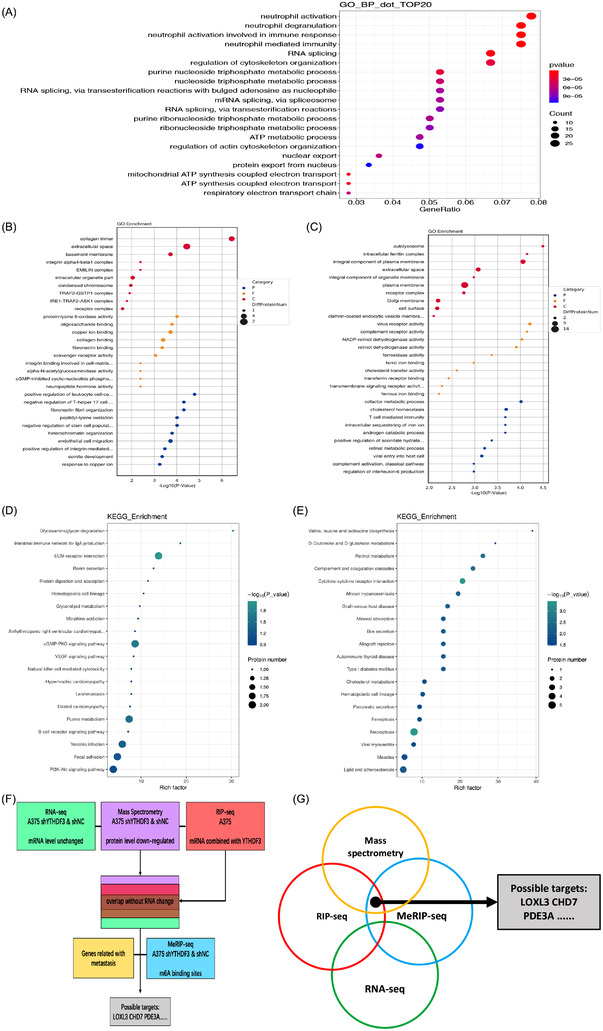
Potential molecules targeted by YTHDF3 in melanoma cells. (A) GO analysis of mRNAs enriched by YTHDF3 in RIP‐seq. GO analysis of (B) downregulated and (C) upregulated differentially expressed proteins identified by mass spectrometry analysis between shNC group and shYTHDF3 group. KEGG analysis of (D) downregulated and (E) upregulated proteins differentially expressed proteins identified by mass spectrometry analysis between shNC group and shYTHDF3 group. (F,G) Overlap of genes identified by RNA‐seq, MeRIP‐seq, RIP‐seq and mass spectrometry analysis.

To find out the targets regulated by YTHDF3, RNA‐seq, MeRIP‐seq, RIP‐seq and mass spectrometry analysis were performed. According to the results of RNA‐seq, we observed that after YTHDF3 downregulation, 382 genes altered globally (*p* < .05 and |log_2_FoldChange|≥1) (Figure 4A). Among these 382 genes, 133 genes were downregulated and 249 were upregulated. The results of KEGG analyses in RNA‐seq showed that genes related to tumour malignant behaviour, extracellular matrix, regulation of cell migration and cell adhesion were enriched (Figure 4B,C). Gene set enrichment analysis (GSEA) showed that the differentially expressed genes after YTHDH3 downregulation were associated with angiogenesis and epithelial mesenchymal transition (Figure 4D,E). These results indicated that YTHDF3 regulated the migration and invasion ability of melanoma cells, and these processes may be associated with the extracellular matrix. Hence, targeting YTHDF3 participating in the process of the extracellular matrix would be promising. To find out the targets enriched with m6A sites and bound with YTHDF3, MeRIP‐seq and RIP‐seq were performed. From the results of MeRIP‐seq performed in shNC and shYTHDF3 of A375 cell, we got 26618 m6A peaks out of 14985 transcripts, and the m6A consensus motif GGAC was identified in both two groups (Figure 4F,G), showing the enrichment of m6A modified RNAs. According to the KEGG analysis of MeRIP‐seq, enriched genes were in focal adhesion, ECM‐receptor interaction, RNA splicing and mRNA processing, and genes in pathways related to cancers were also enriched (Figure 4H,I). Besides, these m6A modifications were predominately enriched in the CDS and 3′UTR regions (Figure 4J–L). The results of RIP‐seq in blank A375 cells revealed that 420 transcripts may be the potential targets of YTHDF3. The terms of cellular components also enriched in items like focal adhesion and adherens junction (Figure 5A).

The previous report has demonstrated that YTHDF3 could regulate the translation efficiency of its targets without affecting its mRNA level.^23^ Therefore, we investigated whether a similar condition exists in melanoma. In order to find targets with decreased protein levels more specifically, mass spectrometry analysis in shNC and shYTHDF3 of A375 cells was carried out. Terms of GO analysis (Figure 5B,C) and KEGG analysis (Figure 5D,E) related that the differentially expressed proteins were enriched in terms associated with ECM and cell junction.

We focused on the transcripts which were enriched with m6A sites and bound with YTHDF3 according to MeRIP‐seq and RIP‐seq. Among the overlap, we eliminated transcripts that were differentially expressed among RNA‐seq. Besides, the decreased protein was included. After screening, we selected multiple targets and finally, the overlap between these results was confirmed with three transcripts, namely, LOXL3, CHD7 and PDE3A (Figure 5F,G).

### 3.5 YTHDF3 regulates translation of LOXL3 in a m6A‐dependent manner

**FIGURE 6:**
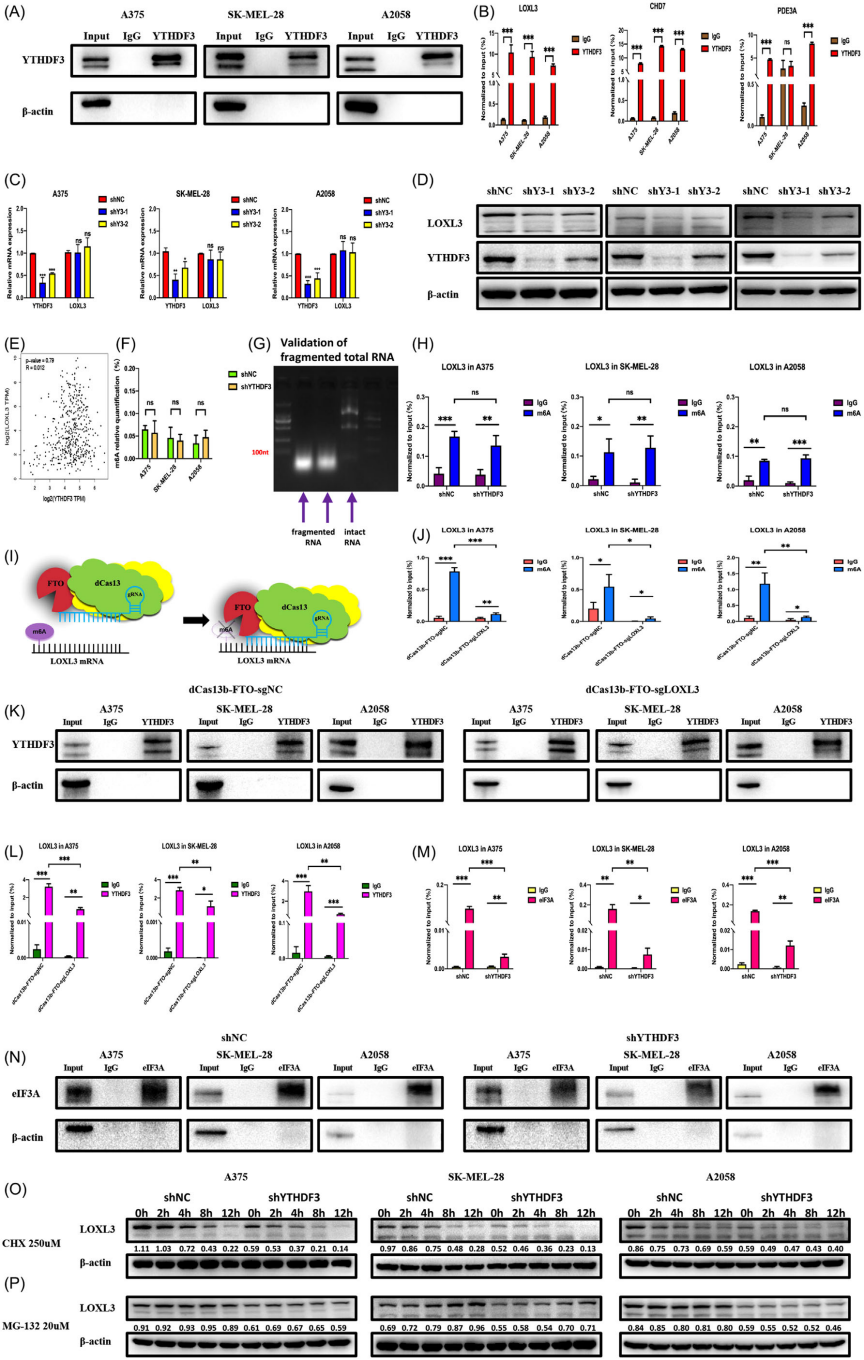
YTHDF3 recognizes and regulates LOXL3 in an m6A‐dependent manner. (A,B) RIP‐qPCR showed the interaction between YTHDF3 protein and mRNA of LOXL3, CHD7 and PDE3A in A375, SK‐MEL‐28 and A2058 melanoma cells. (C,D) mRNA and protein level of LOXL3 after the downregulation of YTHDF3 by RT‐qPCR and Western blot. (E) mRNA level of LOXL3 was not correlated with that of YTHDF3 from the Pearson correlation analysis in the GEPIA dataset. (F) Global quantification of m6A in shNC and shYTHDF3 of A375, SK‐MEL‐28 and A2058 cells. (G,H) Total RNA was well fragmented and m6A level of LOXL3 was detected by MeRIP‐qPCR. (I) Schematic diagram of dCas13b‐FTO‐sgRNA technology. (J) m6A level of dCas13b‐FTO‐sgNC group and dCas13b‐FTO‐sgLOXL3 group detected by MeRIP‐qPCR. (K,L) Interaction between YTHDF3 protein and LOXL3 mRNA in dCas13b‐FTO‐sgNC group and dCas13b‐FTO‐sgLOXL3 group revealed by RIP‐qPCR. (M,N) RIP‐qPCR showed the interaction between eIF3A protein and mRNA of LOXL3 in shNC and shYTHDF3 of A375, SK‐MEL‐28 and A2058 cells. (O) Western blot analysis of LOXL3 protein expression upon CHX treatment in shNC and shYTHDF3 of A375, SK‐MEL‐28 and A2058 cells. (P) Western blot analysis of LOXL3 protein expression upon MG‐132 treatment in shNC and shYTHDF3 of A375, SK‐MEL‐28 and A2058 cells. *, ** and *** means p < .05, *p* < 0.01 and *p* < .001, respectively and ns indicates not significant (*p* > .05).

Based on the literature, LOXL3, CHD7 and PDE3A could regulate the malignant behaviour of tumours. Using RIP‐qPCR, we further confirmed that YTHDF3 could catch these transcripts except PDE3A in SK‐MEL‐28 melanoma cells (Figure 6A,B). Next, we evaluated the expression of LOXL3 which was associated with the extracellular matrix. We observed that after the downregulation of YTHDF3 (shYTHDF3‐1 and shYTHDF3‐2) both in mRNA and protein levels, the protein level of LOXL3 was significantly decreased while its mRNA level was nearly unchanged (Figure 6C,D). Such a phenomenon was verified in three melanoma cell lines including A375, SK‐MEL‐28 and A2058. We found that the mRNA level of LOXL3 was not correlated with that of YTHDF3 from Pearson correlation analysis in the GEPIA dataset (Figure 6E). Besides, we also detected the mRNA and protein levels of CHD7 and PDE3A (Figure S2A,B), and the expression pattern of CHD7 was similar to LOXL3. The discrepancy of PDE3A may be caused by rare m6A sites on its transcript. The IGV results of these three transcripts were also carried out (Figure S2C–E). We speculated that YTHDF3 could influence the translation process of LOXL3, and such a process may be associated with m6A sites. We then investigated the expression of common methyltransferases and demethylases like METTL3, METTL14, FTO and ALKBH5 in three melanoma cell lines (Figure S2F), which demonstrated that the process of m6A modification was available in melanoma cell lines. We quantified the global m6A level in the cell lines (A375, SK‐MEL‐28, and A2058) with or without YTHDF3 downregulation, and observed that the global m6A level was unchanged after knocking down the expression of YTHDF3 (Figure 6F), which was in accordance with previous reports.^24^ What is more, by MeRIP‐qPCR, we also observed that the m6A level of LOXL3 was not significantly changed between shNC and shYTHDF3 groups in those three melanoma cell lines (Figure 6G,H). These results were in accordance with the above mentioned MeRIP‐seq showing that the |log_2_Fold Change| of m6A level in LOXL3 was less than 1.

Furthermore, to verify that the interaction and the subsequent regulatory loop between YTHDF3 and LOXL3 was relied on the m6A sites of LOXL3, with the application of CRISPR/Cas13 engineering technology, we obtained a fusion protein with a combination of catalytically dead Type VI‐B Cas13 enzyme (dCas13) and m6A demethylase FTO (Figure 6I).The sgRNA was designed to specifically target the m6A sites of LOXL3. We co‐transfected dCas13b‐FTO fusion protein and gRNA (sgNC and sgLOXL3) in A375, SK‐MEL‐28 and A2058 cells. Then, we investigated the m6A level between the dCas13b‐FTO‐sgNC group and the dCas13b‐FTO‐sgLOXL3 group. Compared with the dCas13b‐FTO‐sgNC group, an obvious decrease of m6A level on LOXL3 was observed after transfecting with dCas13b‐FTO and sgLOXL3 (Figure 6J). We further verified whether the connection between YTHDF3 and LOXL3 was disrupted via RIP‐qPCR. As expected, the YTHDF3 could not enrich as much as LOXL3 mRNA in the group of dCas13b‐FTO‐sgLOXL3, yet the dCas13b‐FTO‐sgNC group could still enrich LOXL3 mRNA (Figure 6K,L). Instead of deleted binding sites on readers, we obtained direct evidence of m6A sites and their potential binding and regulatory functions. These results verified that the dCas13b‐FTO‐sgLOXL3 tool could lead to the demethylation of LOXL3 mRNA and the interaction between YTHDF3 protein and LOXL3 mRNA was achieved by m6A sites on LOXL3. Besides, to find out how YTHDF3 could affect the protein level of its target LOXL3, we performed eIF3A‐RIP‐qPCR to examine whether eIF3A was the underlying reason for translation promotion. We noticed that after the downregulation of YTHDF3, the binding ability of eIF3a to LOXL3 transcripts was significantly reduced (Figure 6M,N). This result could be served as evidence that YTHDF3 has an impact on the translation of LOXL3. Besides, protein stability or degradation (treated with CHX) and synthesis (treated with MG‐132) assay in different time points were performed in shNC and shYTHDF3 of A375, SK‐MEL‐28 and A2058 cells. It was found that no obvious difference between these cells was noticed (Figure 6O,P). Besides, an RNA stability assay was also performed. Among the results of the RNA stability assay, we noticed that the mRNA level of LOXL3 was nearly not changed (Figure S2G) at the different time points of shNC and shYTHDF3 groups in A375, SK‐MEL‐28 and A2058 after treatment with actinomycin D. Yet, the mRNA level of YTHDF3 was decreased in a time‐dependent manner after treatment with actinomycin D (Figure S2H), which could serve as a positive control. Therefore, it was surprising that the mRNA level of LOXL3 was not influenced by actinomycin D in a time‐dependent manner.

Furthermore, we also observed the expression of LOXL3 after the downregulation of METTL3, a methyltransferase which influenced the m6A sites of LOXL3. We found a discrepancy among A375, SK‐MEL‐28 and A2058 cells. The mRNA level of LOXL3 was unchanged among shNC and shMETTL3 groups in all three cell lines (Figure S2I) and the mRNA level of YTHDF3 was unchanged in A375 and A2058 cells but increased in SK‐MEL‐28 cells. In terms of protein level (Figure S2J), the expression of YTHDF3 and LOXL3 was nearly not changed in A375 cells. In A375 cells, the band of LOXL3 isoform was decreased after the downregulation of METTL3. In SK‐MEL‐28 cells, the expression of YTHDF3 and LOXL3 was all downregulated after the downregulation of METTL3. In A2058 cells, the expression of YTHDF3 was unchanged while the expression of LOXL3 was decreased.

### 3.6 LOXL3 regulates migration and invasion of melanoma

**FIGURE 7:**
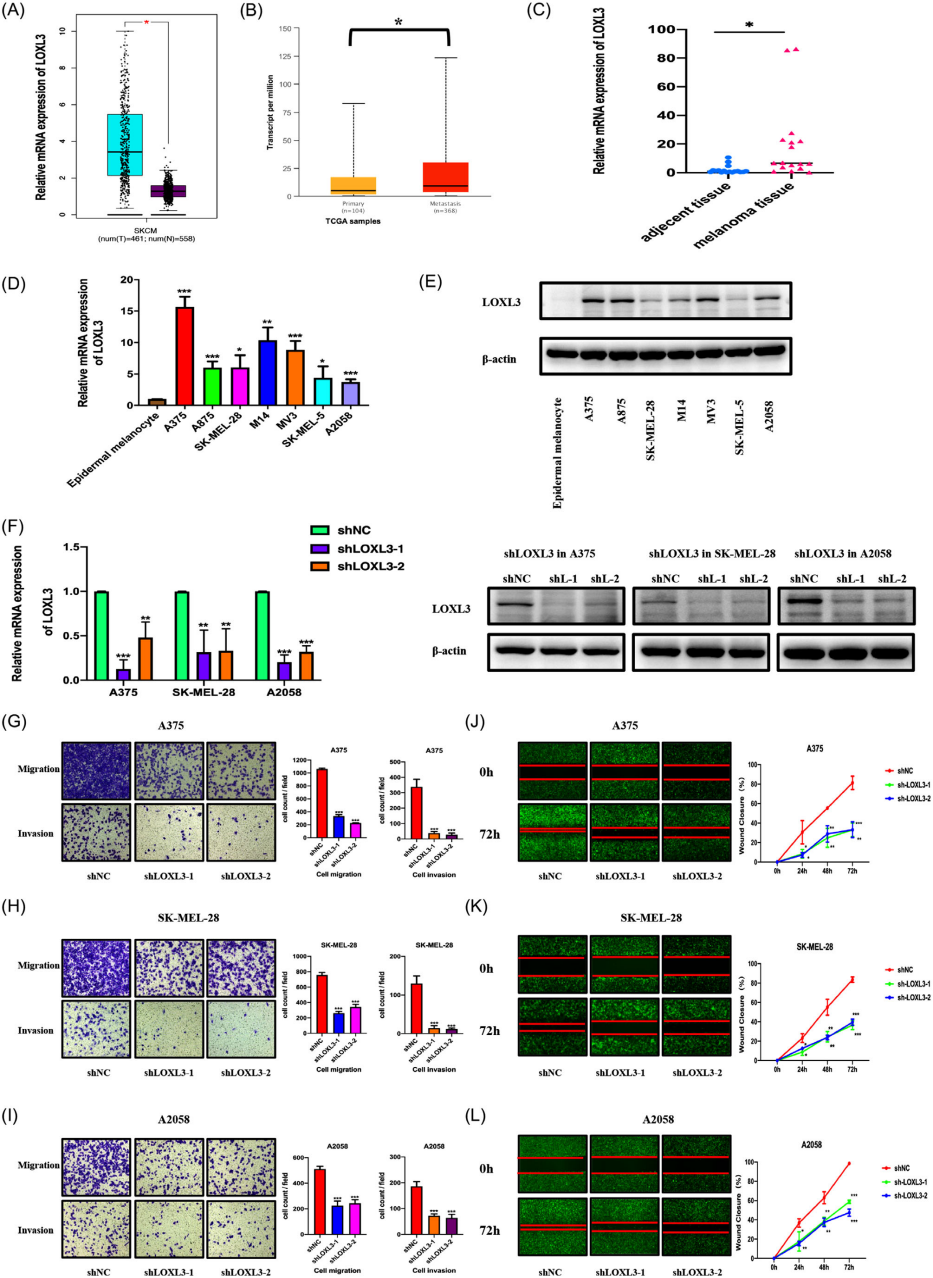
Downregulation of LOXL3 inhibits migration and invasion of melanoma. (A) mRNA expression level of LOXL3 in melanoma tissues (left, blue bar) and normal tissues (right, purple bar) in GEPIA dataset. (B) mRNA expression of LOXL3 in primary melanoma and metastatic melanoma in UALCAN dataset. (C) mRNA expression of LOXL3 is higher in melanoma tissue than that in adjacent tissue (*n* = 17). (D,E) LOXL3 mRNA and protein level in primary epidermal melanocyte and melanoma cell lines (A375, A875, SK‐MEL‐28, M14, MV3, SK‐MEL‐5 and A2058) verified by RT‐qPCR and Western blot. (F) A375, SK‐MEL‐28, A2058 were transfected with two shRNAs targeting LOXL3 and a negative control shRNA. Expression of LOXL3 mRNA and protein detected by RT‐qPCR and Western blot. (G–I) Transwell assay showed that LOXL3 downregulation inhibited migration and invasion ability of A375, SK‐MEL‐28 and A2058 melanoma cells. (J–L) Wound healing experiment showed that LOXL3 downregulation inhibited migration ability of A375, SK‐MEL‐28 and A2058 melanoma cells at 24, 48, 72. *, ** and *** means *p* < .05, *p* < .01 and *p* < .001, respectively and ns indicates not significant (*p* >.05).

The above results suggested that the oncogenic role of YTHDF3 was dependent on LOXL3 since the oncogenic function of LOXL3 had already been reported in melanoma.^25^ Hence, we intended to identify the expression and oncogenic role of LOXL3 in melanoma cells. Online bioinformatic tools including GEPIA and UALCAN database indicated that LOXL3 was overexpressed in melanoma, especially metastatic melanoma (Figure 7A,B). We verified the expression level of mRNA and protein in melanoma tissues (Figure 7C) and melanoma cell lines like A375, A875, SK‐MEL‐28, M14, MV3, SK‐MEL‐5 and A2058 (Figure 7D,E). Similar to the expression of YTHDF3, an increased mRNA and protein level of LOXL3 in melanoma cells compared with epidermal melanocytes.

**FIGURE 8:**
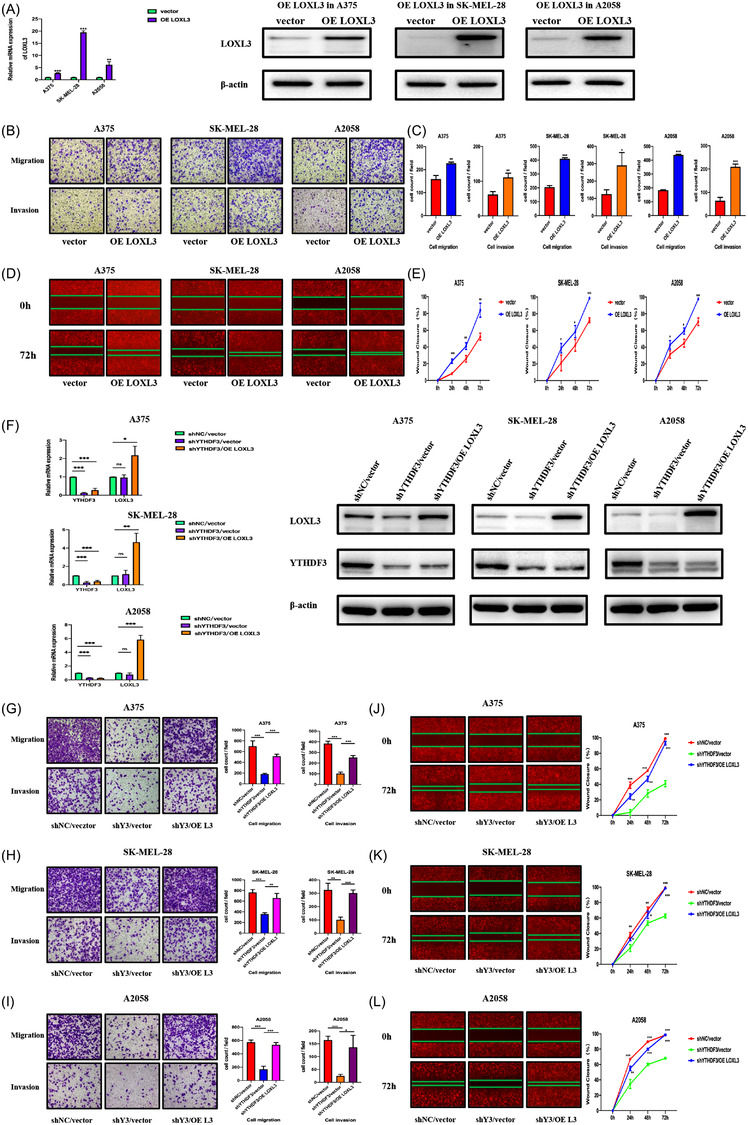
Overexpression of LOXL3 promotes migration and invasion of melanoma. (A) A375, SK‐MEL‐28, A2058 were transfected with lentivirus overexpressing LOXL3 and a blank vector. Expression of LOXL3 mRNA and protein detected by RT‐qPCR and Western blot. (B,C) Transwell assay showing LOXL3 overexpression promoted migration and invasion ability of A375, SK‐MEL‐28 and A2058 melanoma cells. (D,E) Wound healing experiment showing LOXL3 overexpression promoted migration ability of A375, SK‐MEL‐28 and A2058 melanoma cells at 24, 48, 72 and 96 h. (F) In YTHDF3‐ downregulation A375, SK‐MEL‐28 and A2058 melanoma cells, LOXL3 was overexpressed. mRNA and protein expression level of YTHDF3 and LOXL3 were detected by RT‐qPCR and Western blot. (G–I) Transwell assay showing LOXL3 overexpression in YTHDF3‐ downregulation rescued migration and invasion ability of A375, SK‐MEL‐28 and A2058 melanoma cells. (J–L) Wound healing experiment showing LOXL3 overexpression in YTHDF3‐ downregulation rescued migration ability of A375, SK‐MEL‐28 and A2058 melanoma cells at 24, 48, 72. *, ** and *** means *p* < .05, *p* < .01 and *p* < .001, respectively and ns indicates not significant (*p* > .05).

With the assistance of lentivirus, we constructed LOXL3‐downregulation and overexpression A375, SK‐MEL‐28 and A2058 melanoma cells (shLOXL3‐1, shLOXL3‐2 and OE LOXL3). The efficiency of downregulation was determined by RT‐qPCR and Western blot (Figures 7F and 8A). Downregulation of LOXL3 achieved a remarkable inhibitory effect on the migration and invasion of melanoma cells which were observed via transwell assay (Figure 7G–I) and wound healing experiment (Figure 7J–L), while overexpression of LOXL3 could promote the migration and invasion of melanoma cells observed via transwell assay (Figure 8B,C) and wound healing experiment (Figure 8D,E). These results demonstrated that LOXL3 promoted the migration and invasion of melanoma cells and the function of YTHDF3 may depend on LOXL3.

### 3.7 Overexpression of LOXL3 reverses the inhibitory effect of migration and invasion in melanoma caused by YTHDF3 knockdown

**FIGURE 9:**
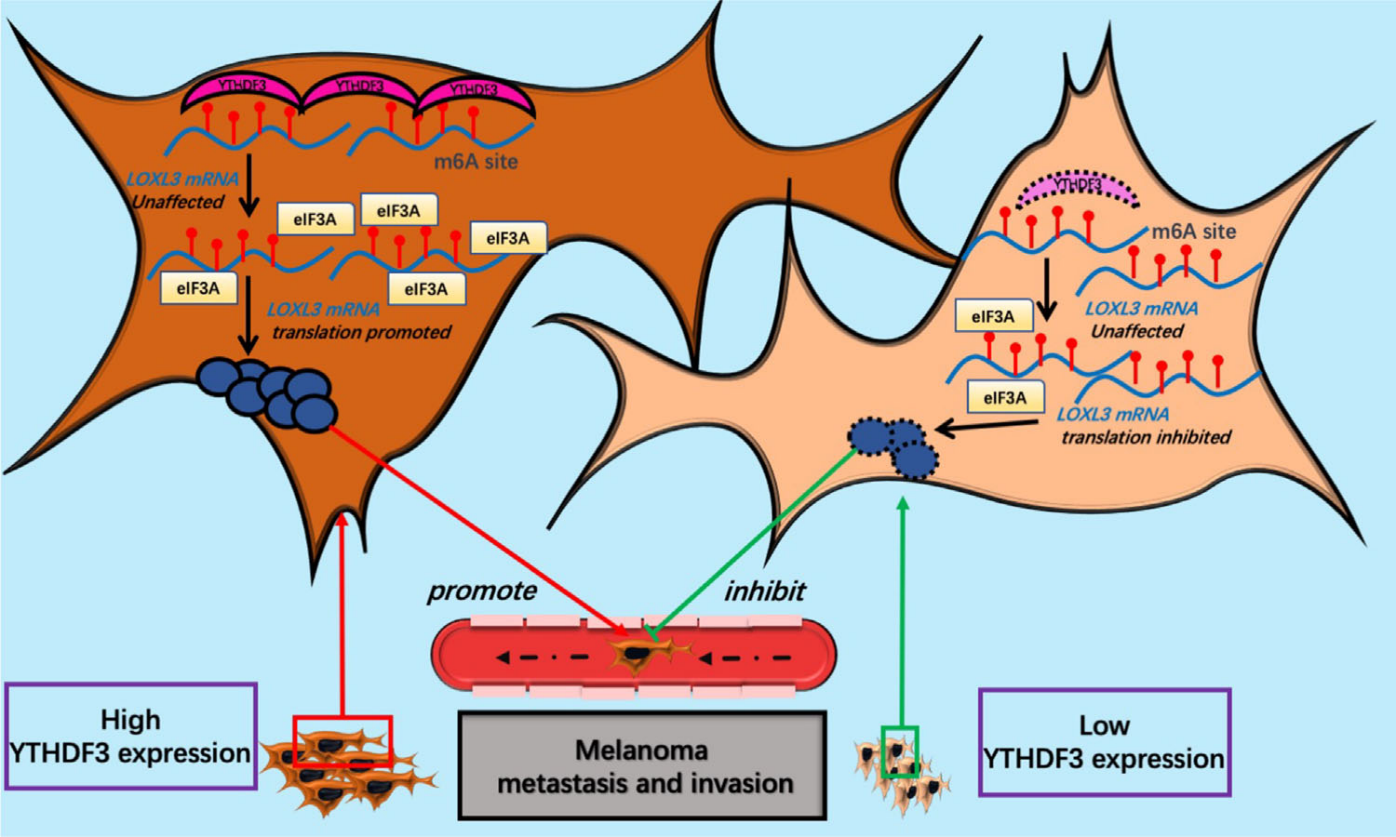
Schematic diagram of YTHDF3 regulating its ‘executor’ LOXL3 in melanoma.

To better understand the role of LOXL3 and its function regulated by YTHDF3, we reversed the expression of LOXL3 in YTHDF3‐ downregulation (shYTHDF3‐1) A375, SK‐MEL‐28 and A2058 melanoma cells. We transfected vector lentivirus to the shNC and shYTHDF3 cells and also transfected lentivirus of LOXL3 overexpression to other shYTHDF3 cells. Three cell groups named shNC/vector, shYTHDF3/vector and shYTHDF3/oeLOXL3 were established. The efficiency of overexpression of LOXL3 was also determined by RT‐qPCR and Western blot (Figure 8F). It was found that after reversing the expression of LOXL3, the migration and invasion of YTHDF3‐downregulation A375, SK‐MEL‐28 and A2058 melanoma cells were recovered to some extent, which were identified by transwell assay (Figure 8G–I) and wound healing experiment (Figure 8J–L). Collectively, these results demonstrated that via its m6A binding sites, LOXL3 not only had an oncogenic role in the migration and invasion of melanoma but served as an important ‘executor’ of YTHDF3 (Figure 9).

## 4 DISCUSSION

Melanoma is the most malignant skin tumour which showed high invasive and metastatic potential and poor prognosis.^26^ With the advance of m6A related technology in recent years, research associated with m6A has been performed, especially in the field of oncology. m6A is a common methylation modification in eukaryotic cells, which affects cell differentiation and tissue development by affecting RNA processing, translation and degradation. Modification of m6A can be achieved by ‘writer’ methyltransferase, ‘eraser’ demethylase and ‘reader’ methylation recognition proteins.^7^ Common methylation enzymes include METTL3/14 and WTAP, demethylation enzymes include FTO and ALKBH5, and methylation recognition enzymes include YTHDF1‐3 and YTHDC1‐2.^7^ At present, it has been revealed that the m6A level and its related proteins can regulate the biological behaviour of tumours. Compared with m6A writers or erasers, ‘reader’ might play a more direct and robust regulatory effect on biological processes.

Previous studies have suggested that m6A‐related proteins are associated with the progression of melanoma. For example, m6A modification of related mRNA can be inhibited by knockout of methyltransferase METTL3/14, and the production of CD8 positive T cells, IFN‐γ, CXCL‐9 and CXCL‐10 in the tumour microenvironment can be increased through IFN‐γ‐STAT1‐IRF1 signalling pathway, thus enhancing the effect of anti‐PD‐1 therapy in the treatment of melanoma.^11^ Similarly, inhibition of ALKBH5 can make melanoma more sensitive to immunotherapy by regulating the expression of Mct4/Slc16a3, the lactose content and Treg cell number in tumour microenvironment.^27^ In addition, FTO knockdown can increase the level of m6A in melanoma cells, which leads to the increase of YTHDF2 and related mRNA decay by affecting PDCD1, CXCR4 and SOX10.^13^ Downregulation of FTO could induce melanoma cells more sensitive to IFN‐γ and more susceptible to PD‐1 treatment.^13^ In conjunctival melanoma, METTL3‐mediated m6A methylation can regulate the cell proliferation, cell cycle, invasion and metastasis of melanoma cells by affecting C‐Met.^16^ Similarly, tissues of conjunctival melanoma typically exhibit low m6A levels, which may be associated with poor prognosis, and such phenomenon may be caused by the methylation‐promoting translation of HINT2 mRNA influenced by YTHDF1.^12^ At present, research on m6A in the field of melanoma mainly focused on methyltransferase and demethylase. Yet, the function of methylation recognition protein in melanoma pathogenesis is still elusive. Among the ‘readers’, there were numerous studies on the function of YTHDF1 and YTHDF2. YTHDF1 was mostly involved in the process of translation regulation while YTHDF2 generally affected the RNA metabolism.^24^ Unlike YTHDF1 and YTHDF2, YTHDF3 may serve as a co‐operator of YTHDF1 and YTHDF2 to influence the translation and decay of m6A enriched mRNAs.^24^ But some articles^18^, ^23^, ^28^, ^29^, ^30^, ^31^ in the different field also hinted us that YTHDF3 could be a potent regulator. Via various results of public datasets, we noticed that in the GEPIA dataset, only YTHDF3 had a significantly higher expression in melanoma tissues. In GEO datasets, except YTHDC2, other m6A readers like YTHDF1, YTHDF2, YTHDF3 and YTHDC1 have a significant higher expression in melanoma tissues. Yet, the expression pattern among primary melanoma and metastatic melanoma had a discrepancy in different datasets. We consistently noticed that YTHDF3 had a higher expression in melanoma and metastatic melanoma among different datasets. Until now, only one report demonstrated that the YTHDF3‐CTNNB1 axis participated in the tumourigenicity of ocular melanoma.^18^ Therefore, the evidence of whether YTHDF3 could have a more robust or independent regulatory role in melanoma was inadequate. Therefore, we explored the methylation recognition protein YTHDF3 and its underlying mechanism in the biological behaviour of melanoma.

By searching on some online bioinformatic websites and previous reports, we found that YTHDF3 was highly expressed in melanoma tissues compared with para‐tumour tissues and benign nevi. Compared with primary melanoma, YTHDF3 was highly expressed in metastatic or invasive melanoma. These findings suggested that YTHDF3 may play a role in affecting the metastasis of melanoma. Currently, various reports have claimed that YTHDF3 may take part in the biological process of migration and invasion. It has been reported that YTHDF3 can inhibit the expression of IGF1R mRNA by enhancing the degradation of IGF1R mRNA, and affect its downstream related molecules such as MMP9, which results in the reduction of the invasion and metastasis ability of trophoblast cells.^31^ In addition, YTHDF3 has been confirmed to be closely associated with brain metastasis of breast cancer instead of lung and bone metastasis of breast cancer. This process may be related to YTHDF3 recognition through the m6A site, which further regulates the protein expression of ST6GALNAC5, GJA1, EGFR and VEGFA.^23^ Hence, influencing the interaction between breast cancer cells and vascular endothelial cells and astrocytes. Meanwhile, a report pointed out that lncRNA GAS5‐YAP‐YTHDF3 could form a feedback loop and participate in the occurrence and development of colorectal cancer.^32^ YAP can promote the expression of YTHDF3, which in turn can promote the degradation of lncRNA by recognizing m6A site on lncGAS5. LncRNA GAS5 can promote the degradation and phosphorylation of YAP, thus inhibiting the occurrence of tumours. Therefore, YTHDF3 also plays the role of an oncogene in colorectal cancer. In hepatocellular carcinoma, circ_KIAA1429 can regulate the invasion, metastasis and epithelial‐mesenchymal transformation of stem cells by affecting Zeb1.^30^ The stability of Zeb1 mRNA can be regulated by YTHDF3's recognition of m6A sites. Hence, we confirmed that YTHDF3 plays a role in regulating melanoma invasion and metastasis both in vitro and in vivo.

Therefore, besides the targets of YTHDF3 revealed by mentioned studies, combining the results of multi‐omics analysis including RNA‐seq, MeRIP‐seq, RIP‐seq and mass spectrometry analysis, we identified several targets regulated by YTHDF3. Since most previous reports claimed that YTHDF3 had a similar function in regulating the process of translation without affecting mRNA levels like YTHDF1,^23^ LOXL3, CHD7 and PDE3A were identified with corresponding standards. Because the terms related to ECM and cell adhesion were frequently shown in the multi‐omics analysis, we investigated whether the above‐mentioned targets were directly participating in these biological processes. Interestingly, lysyl oxidase‐like 3 (LOXL3) belongs to the LOX protein family.^33^ Among these members, it is reported that LOXL3 was involved in various processes, such as embryonic development, maturation of extracellular matrix, tumourigenesis and some fibrous diseases.^33^ In terms of tumours, some reports have pointed out that LOXL3 could affect the migration and invasion of tumour cells, and downregulation of LOXL3 could inhibit the ability of migration and invasion in tumour cells.^25^, ^34^, ^35^, ^36^ The processes involved with LOXL3 may have a close relationship with tumour invasion and metastasis. LOXL3 could augment the process of EMT via enhancing the expression of Snail 1 in hepatocellular carcinoma.^37^ In glioma, researchers noticed that LOXL3 could modulate various molecules related to focal adhesion and cytoskeleton assemblies such as TLN2, ENAH and CCN2.^34^ Processes including cell adhesion complex turnover in glioma were also inhibited after the downregulation of LOXL3.^34^ Besides, molecules including MAPK1, STK38 participating in MAPK/ERK pathway and focal adhesion were also inhibited after the downregulation of LOXL3.^34^ In melanoma, previous reports have pointed out that LOXL3 was associated with the genome stability of melanoma cell and hence impact the mitotic process and apoptosis.^38^ What's more, LOXL3 could also regulate the metastasis of melanoma via affecting the expression of MITF, TWIST1, SNAIL1 and PRRX1, which were molecules that involved in EMT process.^25^ A research also unveiled that LOXL3–SNAIL1–PRRX1 axis was a potent regulatory axis in melanoma.^25^ Therefore, we further validated the relationship between YTHDF3 and LOXL3, as well as the function of LOXL3. It was revealed that LOXL3 mRNA was enriched with m6A sites and could be enriched by YTHDF3 and its protein level could be regulated by YTHDF3 without affecting its mRNA level. Besides, a previous report claimed that the m6A level of most mRNAs increased after YTHDFs were depleted.^24^ Indeed, in our study, we found that the global m6A level was unchanged after the downregulation of YTHDF3 in A375, SK‐MEL28, A2058 cells. Such phenomenon indicated that YTHDF3 perhaps had a more robust regulatory role in promoting the translation of targeted m6A‐enriched mRNAs, instead of affecting its mRNA level. Similarly, we also observed that the downregulation of YTHDF3 did not influence the m6A status of LOXL3 via MeRIP‐qPCR, which at least suggested that YTHDF3 might not have a main effect on the RNA level of LOXL3. Furthermore, a CRISPR–Cas13b‐based epitranscriptome engineering tool was employed to find more direct clues supporting the hypothesis that YTHDF3 binds to LOXL3 mRNA via its m6A sites. Similar technology had been used in the manipulation of m6A RNA modification. Cas9 and Cas12 were generally used for DNA manipulation, yet Cas13‐containing type VI systems were the exclusive CRISPR‐Cas system to cleave ssRNA.^39^ The type VI systems could be divided into four subtypes, namely, VI‐A (Cas13a), VI‐B (Cas13b1/2), VI‐C(Cas13c), VI‐D (Cas13d).^39^ Among them, VI‐B (Cas13b1/2) was the most frequently used tool in m6A modification. For instance, Li J et al.^40^ combined engineered dCas13b (catalytically inactivated Cas13b)‐ALKBH5 fusion protein with sgRNA, which was named the dm6ACRISPR system. Such a system could demethylate m6A sites in targeted mRNA, which could serve as a promising tool in the manipulation of m6A methylation on specific endogenous mRNA. What's more, another system called the targeted RNA methylation (TRM) system was also employed to achieve the m6A installation in endogenous RNA transcripts.^41^ Such system included dCas13, METTL3 without zinc finger RNA‐binding motifs, METTL14 without METTL3‐interacting domain and a Cas13 guide RNA. All these above‐mentioned technologies could achieve a high specificity and low off‐target effect. Like the dm6ACRISPR system, a similar system was designed including a combination of catalytically dead Type VI‐B Cas13 enzyme (dCas13) and m6A demethylase FTO and sgRNA, as well as a sgRNA designed to specifically target the m6A sites of LOXL3. We noticed that compared with the dCas13b‐FTO‐sgNC group, the m6A level of LOXL3 mRNA and the bond between YTHDF3 and LOXL3 mRNA were both decreased significantly. Unlike previous studies^42^ that mainly focus on the binding sites of m6A readers, our results may provide more direct evidence showing that m6A sites were indeed the connection site between YTHDF3 and its targets such as LOXL3 mRNA. To find out the underlying mechanism of translation promotion of LOXL3 caused by YTHDF3, we collected evidence from different aspects. It was reported that YTHDF3 could influence the RNA stability, similar to YTHDF2,^24^ and promote the RNA stability of targeted mRNA.^28^, ^29^ Our results indicated that the mRNA stability of LOXL3 was not affected by YTHDF3. Interestingly, we noticed that the mRNA level of LOXL3 was nearly not changed at the different time points of shNC and shYTHDF3 groups in A375, SK‐MEL‐28 and A2058 cells after treatment with actinomycin D until cells were mostly dead. We changed different primers of LOXL3 and the results were identical. To exclude the possibility of unexpected or subjective errors, we also detected the mRNA level of YTHDF3 as the positive control. The mRNA level of YTHDF3 was decreased in a time‐dependent manner after treatment with actinomycin D. Therefore, it was strange that the mRNA level of LOXL3 was not influenced by actinomycin D in a time‐dependent manner. The results that the RNA level of LOXL3 was unchanged after treated with actinomycin D could demonstrate that the influence of YTHDF3 on LOXL3 may not have occurred at the RNA level.

Hence, the process of translation should be focused on. Experiments including protein stability/degradation and synthesis assay were carried out to show the change of protein expression of LOXL3 after YTHDF3 downregulation, which was not related to ubiquitination. The results of no change among shNC and shYTHDF3 groups treated with CHX and MG‐132 suggested that there might be some other degradation mechanisms. Therefore, the accumulation of LOXL3 protein was not obvious as time went by. We consequently focus on the origin of protein production. Additionally, we found that the process of LOXL3 translation promotion caused by YTHDF3 was associated with eIF3A, which was in accordance with previous reports.^23^, ^43^ To better show the translation promotion between YTHDF3 and LOXL3 was related to m6A sites, we examined the expression of RNA and protein level of YTHDF3 and LOXL3 after METTL3 downregulation, which may disturb the m6A abundance of LOXL3 transcript. A discrepancy occurred in three cell lines perhaps caused by some other underlying regulatory mechanisms. Yet, results that the RNA level of LOXL3 was unchanged after METTL3 downregulation, combined with the results of RNA stability, could serve as evidence that YTHDF3 may not influence the RNA level of LOXL3. Finally, we confirmed that LOXL3 has a high expression level in both RNA and protein levels, and plays a role in the migration and invasion of melanoma cells. Downregulation of LOXL3 inhibits the migration and invasion of melanoma cells and vice versa. LOXL3 is a downstream target of YTHDH3 and re‐expression of LOXL3 reverses its inhibitory effects in migration and invasion of melanoma cells.

Collectively, our work revealed the regulatory role of YTHDF3 in the migration, invasion and metastasis of melanoma cells both in vitro and in vivo. This function is related to LOXL3, a potential ‘executor’ of YTHDF3. Mechanistically, YTHDF3 recognizes the m6A sites on LOXL3 and promotes its protein expression without affecting its mRNA level via the enrichment of eIF3A on the transcript of LOXL3. YTHDF3 and its downstream LOXL3 play a critical role in melanoma metastasis and could serve as a potential therapeutic target to be intervened.

## CONFLICT OF INTEREST

The authors declare no conflict of interest.

## Supporting information



Figure S1 Expression of YTHDF1, YTHDF2, YTHDC1 and YTHDC2 in melanoma.

Figure S2 Expression of other targets of YTHDF3 and the expression of YTHDF3 or LOXL3 after different situations.

## References

[1] Yang Y , Hsu PJ , Chen YS , Yang YG . Dynamic transcriptomic m(6)A decoration: writers, erasers, readers and functions in RNA metabolism. Cell Res. 2018;28:616‐624. 10.1038/s41422-018-0040-8 29789545 PMC5993786

[2] Li M , Zha X , Wang S . The role of N6‐methyladenosine mRNA in the tumor microenvironment. Biochim Biophys Acta Rev Cancer. 2021;1875:188522. 10.1016/j.bbcan.2021.188522 33545295

[3] Wang T , Kong S , Tao M , Ju S . The potential role of RNA N6‐methyladenosine in cancer progression. Mol Cancer. 2020;19:88. 10.1186/s12943-020-01204-7 32398132 PMC7216508

[4] Dai D , Wang H , Zhu L , Jin H , Wang X . N6‐methyladenosine links RNA metabolism to cancer progression. Cell Death Dis. 2018;9:124. 10.1038/s41419-017-0129-x 29374143 PMC5833385

[5] Ma Z , Ji J . N6‐methyladenosine (m6A) RNA modification in cancer stem cells. Stem Cells. 2020;38(12):1511‐1519. 10.1002/stem.3279 32985068

[6] Zhou Z , Lv J , Yu H , et al. Mechanism of RNA modification N6‐methyladenosine in human cancer. Mol Cancer. 2020;19:104. 10.1186/s12943-020-01216-3 32513173 PMC7278081

[7] Deng LJ , Deng W‐Q , Fan S‐R , et al. m6A modification: recent advances, anticancer targeted drug discovery and beyond. Mol Cancer. 2022;21:52. 10.1186/s12943-022-01510-2 35164788 PMC8842557

[8] Hao L , Yin J , Yang H , et al. ALKBH5‐mediated m(6)A demethylation of FOXM1 mRNA promotes progression of uveal melanoma. Aging. 2021;13:4045‐4062. 10.18632/aging.202371 33428593 PMC7906204

[9] Liu J , Zhou Z , Ma L , et al. Effects of RNA methylation N6‐methyladenosine regulators on malignant progression and prognosis of melanoma. Cancer Cell Int. 2021;21:453. 10.1186/s12935-021-02163-9 34446007 PMC8393813

[10] Feng ZY , Wang T , Su X , Guo S . Identification of the m(6)A RNA methylation regulators WTAP as a novel prognostic biomarker and genomic alterations in cutaneous melanoma. Front Mol Biosci. 2021;8:665222. 10.3389/fmolb.2021.665222 34291082 PMC8287526

[11] Wang L , Hui H , Agrawal K , et al. m(6)A RNA methyltransferases METTL3/14 regulate immune responses to anti‐PD‐1 therapy. EMBO J. 2020;39:e104514. 10.15252/embj.2020104514 32964498 PMC7560214

[12] Jia R , Chai P , Wang S , et al. m(6)A modification suppresses ocular melanoma through modulating HINT2 mRNA translation. Mol Cancer. 2019;18:161. 10.1186/s12943-019-1088-x 31722709 PMC6854757

[13] Yang S , Wei J , Cui Y‐H , et al. m(6)A mRNA demethylase FTO regulates melanoma tumorigenicity and response to anti‐PD‐1 blockade. Nat Commun. 2019;10:2782. 10.1038/s41467-019-10669-0 31239444 PMC6592937

[14] Bhattarai PY , Kim G , Poudel M , Lim SC , Choi HS . METTL3 induces PLX4032 resistance in melanoma by promoting m(6)A‐dependent EGFR translation. Cancer Lett. 2021;522:44‐56. 10.1016/j.canlet.2021.09.015 34530048

[15] Wu H , Xu H , Jia D , Li T , Xia L . METTL3‐induced UCK2 m(6)A hypermethylation promotes melanoma cancer cell metastasis via the WNT/β‐catenin pathway. Ann Transl Med. 2021;9:1155. 10.21037/atm-21-2906 34430596 PMC8350655

[16] Luo G , Xu W , Zhao Y , et al. RNA m(6)A methylation regulates uveal melanoma cell proliferation, migration, and invasion by targeting c‐Met. J Cell Physiol. 2020;235:7107‐7119. 10.1002/jcp.29608 32017066

[17] Dahal U , Le K , Gupta M . RNA m6A methyltransferase METTL3 regulates invasiveness of melanoma cells by matrix metallopeptidase 2. Melanoma Res. 2019;29:382‐389. 10.1097/cmr.0000000000000580 30762711

[18] Xu Y , He X , Wang S , et al. The m(6)A reading protein YTHDF3 potentiates tumorigenicity of cancer stem‐like cells in ocular melanoma through facilitating CTNNB1 translation. Oncogene. 2022;41:1281‐1297. 10.1038/s41388-021-02146-0 35110680

[19] Gutzmer R , Stroyakovskiy D , Gogas H , et al. Atezolizumab, vemurafenib, and cobimetinib as first‐line treatment for unresectable advanced BRAF(V600) mutation‐positive melanoma (IMspire150): primary analysis of the randomised, double‐blind, placebo‐controlled, phase 3 trial. Lancet. 2020;395:1835‐1844. 10.1016/s0140-6736(20)30934-x 32534646

[20] Hugo W , Zaretsky JM , Sun L , et al. Genomic and transcriptomic features of response to anti‐PD‐1 therapy in metastatic melanoma. Cell. 2016;165:35‐44. 10.1016/j.cell.2016.02.065 26997480 PMC4808437

[21] Tang Z , Li C , Kang B , et al. GEPIA: a web server for cancer and normal gene expression profiling and interactive analyses. Nucleic Acids Res. 2017;45:W98‐W102. 10.1093/nar/gkx247 28407145 PMC5570223

[22] Chandrashekar DS , Bashel B , Balasubramanya SAH , et al. UALCAN: a portal for facilitating tumor subgroup gene expression and survival analyses. Neoplasia. 2017;19:649‐658. 10.1016/j.neo.2017.05.002 28732212 PMC5516091

[23] Chang G , Shi L , Ye Y , et al. YTHDF3 induces the translation of m(6)A‐enriched gene transcripts to promote breast cancer brain metastasis. Cancer Cell. 2020;38:857‐871.e857. 10.1016/j.ccell.2020.10.004 33125861 PMC7738369

[24] Shi H , Wang X , Lu Z , et al. YTHDF3 facilitates translation and decay of N(6)‐methyladenosine‐modified RNA. Cell Res. 2017;27:315‐328. 10.1038/cr.2017.15 28106072 PMC5339834

[25] Vázquez‐Naharro A , Bustos‐Tauler J , Floristán A , et al. Loxl3 promotes melanoma progression and dissemination influencing cell plasticity and survival. Cancers. 2022;14(5):1200. 10.3390/cancers14051200 35267510 PMC8909883

[26] Coricovac D , Dehelean C , Moaca E‐A , et al. Cutaneous melanoma‐a long road from experimental models to clinical outcome: a review. Int J Mol Sci. 2018;19(6):1566. 10.3390/ijms19061566 PMC6032347 29795011

[27] Li N , Kang Y , Wang L , et al. ALKBH5 regulates anti‐PD‐1 therapy response by modulating lactate and suppressive immune cell accumulation in tumor microenvironment. Proc Natl Acad Sci U S A. 2020;117:20159‐20170. 10.1073/pnas.1918986117 32747553 PMC7443867

[28] Liao L , He Y , Li S‐J , et al. Anti‐HIV drug elvitegravir suppresses cancer metastasis via increased proteasomal degradation of m6A methyltransferase METTL3. Cancer Res. 2022;82:2444‐2457. 10.1158/0008-5472.Can-21-4124 35507004

[29] Lin Y , Jin X , Nie Q , et al. YTHDF3 facilitates triple‐negative breast cancer progression and metastasis by stabilizing ZEB1 mRNA in an m(6)A‐dependent manner. Ann Transl Med. 2022;10:83. 10.21037/atm-21-6857 35282088 PMC8848410

[30] Wang M , Yang Y , Yang J , Yang J , Han S . circ_KIAA1429 accelerates hepatocellular carcinoma advancement through the mechanism of m(6)A‐YTHDF3‐Zeb1. Life Sci. 2020;257:118082. 10.1016/j.lfs.2020.118082 32653519

[31] Zheng Q , Gan H , Yang F , et al. Cytoplasmic m(1)A reader YTHDF3 inhibits trophoblast invasion by downregulation of m(1)A‐methylated IGF1R. Cell Discov. 2020;6:12. 10.1038/s41421-020-0144-4 32194978 PMC7062805

[32] Ni W , Yao S , Zhou Y , et al. Long noncoding RNA GAS5 inhibits progression of colorectal cancer by interacting with and triggering YAP phosphorylation and degradation and is negatively regulated by the m(6)A reader YTHDF3. Mol Cancer. 2019;18:143. 10.1186/s12943-019-1079-y 31619268 PMC6794841

[33] Ye M , Song Y , Pan S , et al. Evolving roles of lysyl oxidase family in tumorigenesis and cancer therapy. Pharmacol Ther. 2020;215:107633. 10.1016/j.pharmthera.2020.107633 32693113

[34] Laurentino TS , Soares RDS , Lerario AM , Marie SKN , Oba‐Shinjo SM . LOXL3 silencing affected cell adhesion and invasion in U87MG glioma cells. Int J Mol Sci. 2021;22(15):8072. 10.3390/ijms22158072 34360836 PMC8347215

[35] Laurentino TS , Soares RDS , Marie SKN , Oba‐Shinjo SM . LOXL3 function beyond amino oxidase and role in pathologies, including cancer. Int J Mol Sci. 2019; 20. 10.3390/ijms20143587 PMC6678131 31340433

[36] Wang N , Zhou X , Tang F , Wang X , Zhu X . Identification of LOXL3‐associating immune infiltration landscape and prognostic value in hepatocellular carcinoma. Virchows Arch. 2021;479:1153‐1165. 10.1007/s00428-021-03193-4 34448895

[37] Li R , Shang R , Li S , et al. LOXL3‐promoted hepatocellular carcinoma progression via promotion of Snail1/USP4‐mediated epithelial‐mesenchymal transition. Environ Toxicol. 2022;37:2540‐2551. 10.1002/tox.23617 35841383

[38] Santamaría PG , Floristán A , Fontanals‐Cirera B , et al. Lysyl oxidase‐like 3 is required for melanoma cell survival by maintaining genomic stability. Cell Death Differ. 2018;25:935‐950. 10.1038/s41418-017-0030-2 29229995 PMC5907912

[39] Sun X , Wang DO , Wang J . Targeted manipulation of m(6)A RNA modification through CRISPR‐Cas‐based strategies. Methods. 2022;203:56‐61. 10.1016/j.ymeth.2022.03.006 35306148

[40] Li J , Chen Z , Chen F , et al. Targeted mRNA demethylation using an engineered dCas13b‐ALKBH5 fusion protein. Nucleic Acids Res. 2020;48:5684‐5694. 10.1093/nar/gkaa269 32356894 PMC7261189

[41] Wilson C , Chen PJ , Miao Z , Liu DR . Programmable m(6)A modification of cellular RNAs with a Cas13‐directed methyltransferase. Nat Biotechnol. 2020;38:1431‐1440. 10.1038/s41587-020-0572-6 32601430 PMC7718427

[42] Liu T , Wei Q , Jin J , et al. The m6A reader YTHDF1 promotes ovarian cancer progression via augmenting EIF3C translation. Nucleic Acids Res. 2020;48(7):3816‐3831. 10.1093/nar/gkaa048 31996915 PMC7144925

[43] Zhao Y , Zhao H , Zhang D , et al. YTHDF3 facilitates eIF2AK2 and eIF3A recruitment on mRNAs to regulate translational processes in oxaliplatin‐resistant colorectal cancer. ACS Chem Biol. 2022;17:1778‐1788. 10.1021/acschembio.2c00131 35708211

