# Supplementary figures and images for: N6‐methyladenosine reader YTHDF3 regulates melanoma metastasis via its ‘executor'LOXL3
Source: Clin Transl Med. 2022 Nov 2;12(11):e1075. doi: 10.1002/ctm2.1075 (PMC9630608; doi:10.1002/ctm2.1075)

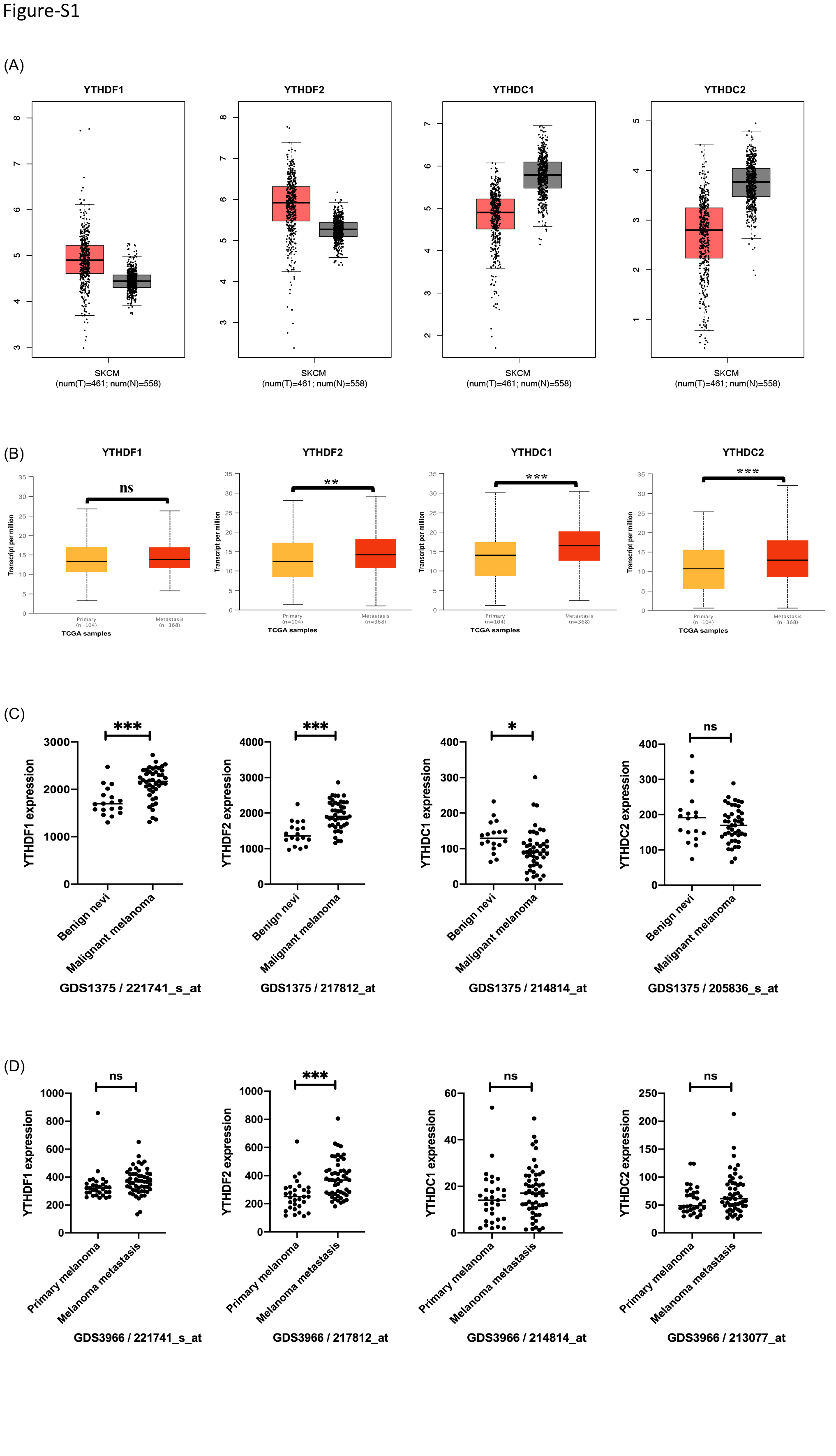

Supplement: Supplementary file 1 — Figure S1 Expression of YTHDF1, YTHDF2, YTHDC1 and YTHDC2 in melanoma. [file CTM2-12-e1075-s001.tif]

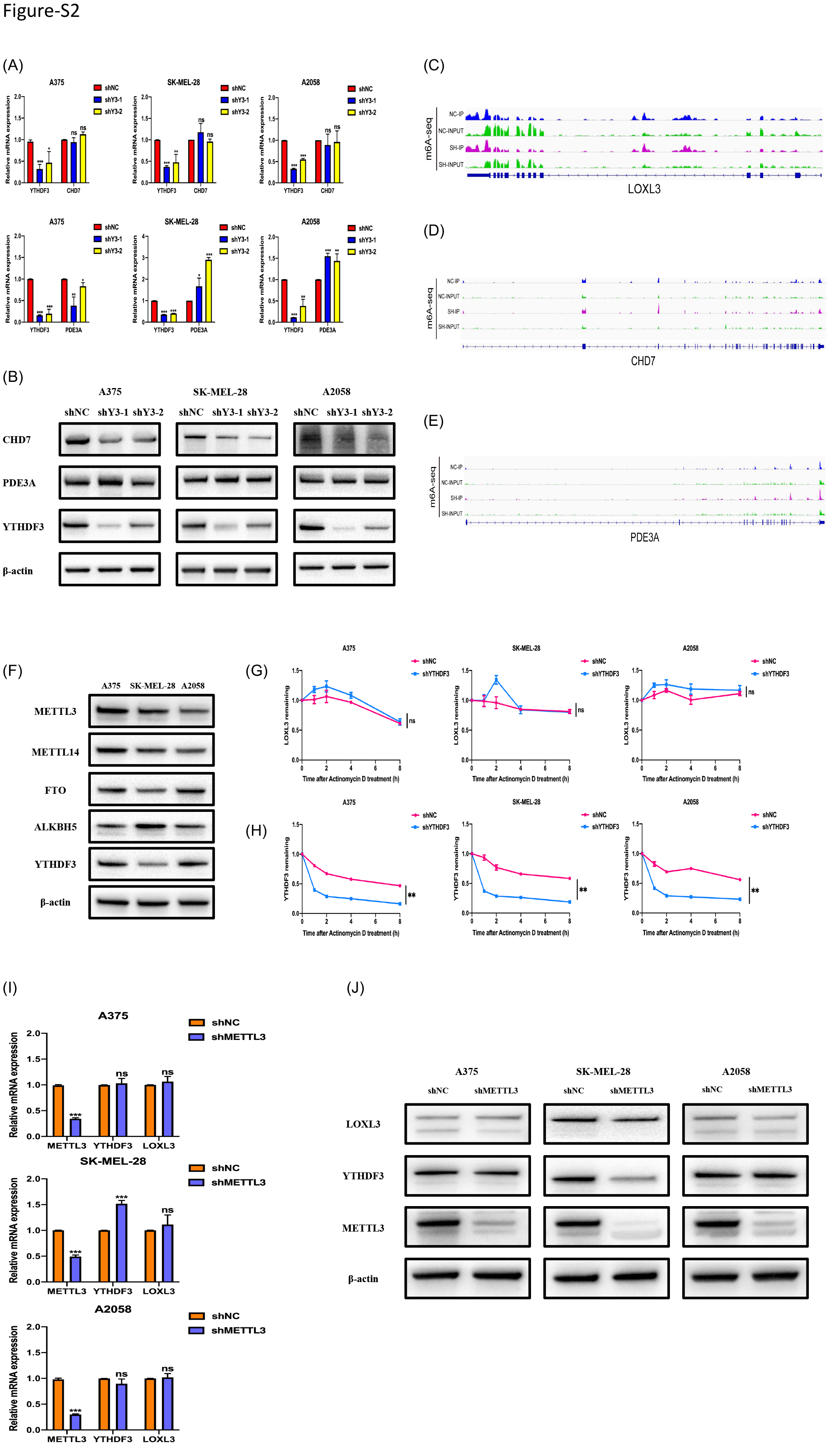

Supplement: Supplementary file 2 — Figure S2 Expression of other targets of YTHDF3 and the expression of YTHDF3 or LOXL3 after different situations. [file CTM2-12-e1075-s002.tif]
